# Pitfalls in spine injuries: risk of dramatic aortic dissection

**DOI:** 10.11604/pamj.2020.37.242.26771

**Published:** 2020-11-16

**Authors:** Giuseppe Rosario Schirò, Pietro Domenico Giorgi

**Affiliations:** 1 Grande Ospedale Metropolitano Niguarda, Milan, Italy

**Keywords:** Spinal fracture, ankylosing spondylitis, trauma

## Image in medicine

A 68 years old man was brought to the resuscitation room of our hospital following a fall from height. During the Focused Assessment with Sonography for Trauma-ultrasound (FAST-US), free liquid in abdomen was found. An angio-computed tomography (CT) (A, B, C) was performed showing a displaced T12 fracture in ankylosing spondylitis (AS) with a dangerous compression on the descending aorta by the displaced anterior wall of the fractured vertebra. This finding suggested high risk of iatrogenic aortic lesion during the surgical reduction of the fracture. Therefore, combined vascular and spinal surgery was deemed necessary. An inflatable balloon was placed endovascularly ready to stop the eventual bleeding and an endovascular prosthesis was ready to be positioned to repair the vascular lesion during the spine surgery. In this way, fracture reduction and fixation were safely performed. Aortic injury associated to thoracic spine fracture in AS is uncommon but potentially fatal. Due to the inflammation and adventitial scarring, the aorta can become tethered to the anterior longitudinal ligament and thus is subjected to shearing forces in an acute trauma. The fracture is generally associated to hyperextension mechanism, largely due to preexisting kyphotic deformity and there is a strong association with unstable B like-chance fractures. Undiagnosed vascular compression could be very dangerous leading to fatal dissection during spine surgery with mortality approaching 60%. In conclusion, spine injuries for vertebral fracture in patient with AS should be considered insidious and at risk of dramatic vascular lesion. Angio-CT and multidisciplinary management should be considered mandatory in cases of displaced fractures.

**Figure 1:**
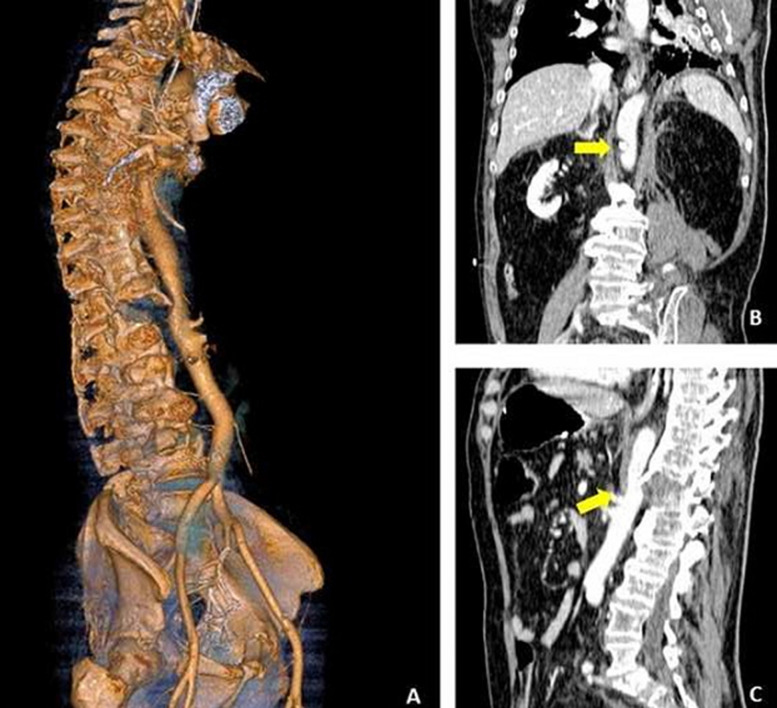
3-D reconstruction showing aortic compression caused by the displaced fragments of an unusual spinal fracture pattern

